# Mapping Morphine’s Antinociceptive Impact on the Ventral Tegmental Area During Nociceptive Stimulation: A Novel Microimaging Approach in a Neuropathic Pain Model

**DOI:** 10.3390/ijms26136526

**Published:** 2025-07-07

**Authors:** Austin Ganaway, Airi Kamata, Dunyan Yao, Kazuto Sakoori, Ryoma Okada, Ting Chen, Yasumi Ohta, Jun Ohta, Masahiro Ohsawa, Metin Akay, Yasemin M. Akay

**Affiliations:** 1 Biomedical Engineering Department, University of Houston, 3517 Cullen Blvd, Houston, TX 77204, USA; austinganaway@gmail.com (A.G.); dunyanyaochris@gmail.com (D.Y.); ting.y.chen@gmail.com (T.C.); makay58@gmail.com (M.A.); 2 Laboratory of Systems Pharmacology, Faculty of Pharmaceutical Sciences, Teikyo University, Tokyo 173-8605, Japanoosawa.masahiro.bg@teikyo-u.ac.jp (M.O.); 3 Division of Materials Science, Graduate School of Science and Technology, Nara Institute of Science and Technology, Ikoma 630-0101, Japan; okada.ryoma.on9@ms.naist.jp (R.O.); ohtay@ms.naist.jp (Y.O.); ohta@ms.naist.jp (J.O.)

**Keywords:** chronic pain, neuropathic pain, partial nerve ligation model, morphine, dopamine, VTA, GABA, CMOS imaging, GCaMP6, fluorescence imaging

## Abstract

The neurobiology of chronic pain is complex and multifaceted, intertwining with the mesocorticolimbic system to regulate the behavioral and perceptional response to adverse stimuli. Specifically, the ventral tegmental area (VTA), the dopaminergic hub of the reward pathways located deep within the midbrain, is crucial for regulating the release of dopamine (DA) throughout the central nervous system (CNS). To better understand the nuances among chronic pain, VTA response, and therapeutics, implementing progressive approaches for mapping and visualizing the deep brain in real time during nociceptive stimulation is crucial. In this study, we utilize a fluorescence imaging platform with a genetically encoded calcium indicator (GCaMP6s) to directly visualize activity in the VTA during acute nociceptive stimulation in both healthy adult mice and adult mice with partial nerve ligation (PNL)-induced neuropathic pain. We also investigate the visualization of the analgesic properties of morphine. Deep brain imaging using our self-fabricated µ-complementary metal–oxide–semiconductor (CMOS) imaging device allows the tracking of the VTA’s response to adverse stimuli. Our findings show that nociceptive stimulation is associated with a reduction in VTA fluorescence activity, supporting the potential of this platform for visualizing pain-related responses in the central nervous system. Additionally, treatment with morphine significantly reduces the neuronal response caused by mechanical stimuli and is observable using the CMOS imaging platform, demonstrating a novel way to potentially assess and treat neuropathic pain.

## 1. Introduction

Chronic pain and its interdependent relationship with one’s sensitivity to acute nociception creates a subjective experience regarding pain perception and variations in neuronal response, making clinical assessment and treatment more complex. Therefore, the ability to visualize the physiological response induced by nociception is crucial to improving the identification and treatment of chronic neuropathic pain symptoms. One such method for visualization resides within the relationship between pain and the mesocorticolimbic system, the dopaminergic pathway in the brain. Within this system lies the ventral tegmental area (VTA), encapsulating a large portion of dopamine (DA) neurons, with the overarching role of interpreting and responding to reward stimuli by modulating the release of DA [1]. Regulation of DA is exerted through a complex and dynamic system with multiple factors at play, including GABAergic interneurons nestled in the VTA, partially responsible for attenuating the DA neuron activity, which acts as a mechanism for maintaining homeostasis [2,3]. Additionally, other regulating factors, such as glutamate’s excitatory relationship with the VTA, can excite DA neurons directly and enhance GABAergic inhibition, influencing the net effect of therapeutic approaches [4]. Introducing our µ-complementary metal–oxide–semiconductor (CMOS) imaging device into the VTA, combined with viral expression of GCaMP6s, allows visualization of the VTA’s activity in real time relating to mechanical hind paw nociceptive stimulation. As the fluorescence activity is modulated, we investigate the complex interplay among acute nociception, chronic pain, and therapeutic treatments in the context of dopaminergic and GABAergic regulation.

**Figure 1:**
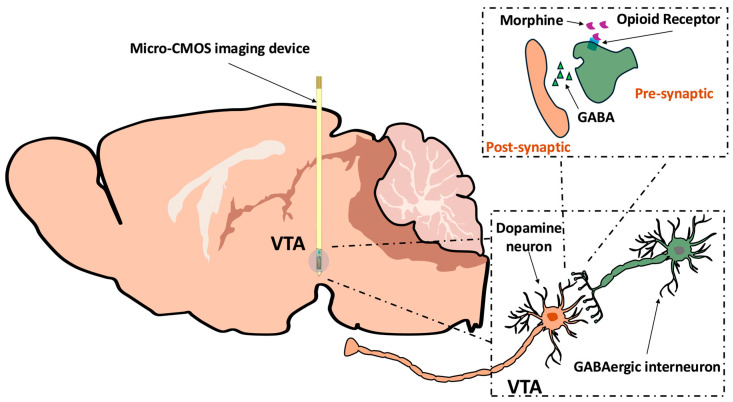
A diagram illustrating how morphine modulates GABAergic and dopaminergic interactions affecting homeostatic regulation of the ventral tegmental area (VTA), adapted from our recent paper [15]. Morphine binds to the presynaptic µ-opioid receptors, reducing the release of GABA into the extracellular space and shifting the balance of inhibitory control over VTA dopamine (DA) neurons. As a result, DA activity increases, facilitating enhanced transmission throughout the reward network. However, the net effect of morphine is not solely due to GABA inhibition but also due to glutamatergic excitatory inputs and other neuromodulatory influences. The placement of the complementary metal–oxide–semiconductor (CMOS) µ-imaging device allows for a vertical viewing plane of the VTA, causing fluorescence to occur due to the µ-LED given the expression of GCaMP6s and detects the fluorescence response that is modulated by the presence of morphine in the extracellular space.

The ability to perceive and interpret noxious stimuli transmitted by the central nervous system (CNS), known as nociception, is a sensory process designed to prevent or minimize injury through nociceptor activation [5,6]. Though not commonly perceived as integral to pain regulation, the VTA assists complex functions regarding the modulation of affective and motivational components of pain via interaction with GABAergic interneurons and ascending neuromodulatory systems [7]. Opioids are frequently prescribed to patients experiencing moderate to severe chronic pain, with the most commonly prescribed drug being morphine [8,9]. An extremely addictive drug with high rates of abuse, morphine binds to the µ-opioid receptors on the presynaptic terminals of the GABAergic interneurons within the VTA, as shown in Figure 1, attenuating the release of GABA and increasing dopaminergic activity [10,11]. This modulation of the VTA introduces an analgesic effect, significantly reducing the patient’s perception of both chronic and acute pain, primarily due to the increased amount of extracellular DA within the mesocorticolimbic pathway [9]. Previous research from our group and collaborators using this imaging platform has investigated crucial pathways regarding both pain and addiction, highlighting its effectiveness in a multitude of potential applications [12,13,14,15,16,17]. Our recent works regarding the VTA showed conclusive evidence that the CMOS imaging platform can reliably detect VTA activity fluorescence increases when the reward pathways are activated due to nicotine exposure in both mice and rats [13,14]. Notably, the intensity of nicotine exposure was directly proportional to the increase in fluorescence activity. Our group previously looked at acute pain response in various brain regions, demonstrating reliability in detecting serotonergic responses with sufficient spatial and temporal resolutions [12]. Serotonin activation of the dorsal raphe nucleus (DRN) following pain stimulation was observed in tandem with microdialysis, showing the efficacy of understanding and mapping pain and its versatility in being paired with other forms of experimentation to yield a more comprehensive understanding of underlying mechanisms [12].

This study evaluates how chronic neuropathic pain alters VTA activity during acute nociceptive stimulation, allowing observation of individual sensitivity and perception of pain, and whether morphine administration assists in reducing VTA activity suppression in freely moving conditions. The foundational efficacy of this platform to visualize the nociceptive response of the VTA is evaluated. We hypothesize that chronic pain will induce a more pronounced decrease in VTA activity upon stimulation. Additionally, we hypothesize that our CMOS imaging platform will allow the visualization of morphine’s analgesic effect by observing the fluorescence response of the VTA to stimuli. By introducing varying mechanical acute sensory forces, we aim to visualize the fluorescence activity response pattern in a healthy mouse model and a partial nerve ligation (PNL) model. Additionally, by introducing antinociceptive conditions derived from morphine administration, underlying biological mechanisms may be validated, and the antinociceptive effect of opioids can be demonstrated in real time. Therefore, mechanical sensory stimulation may be confirmed to be of a nociceptive nature if the presence of neuropathic pain exacerbates the depression in VTA activity. Morphine administration, which alleviates VTA depression, further reinforces the potential correlation between fluorescence signaling in the VTA and the visualization of mechanically induced nociception.

## 2. Results

### 2.1. Sham and Pain Model Mice Responses to Graded Stimulation and Analgesic Condition

**Figure 2:**
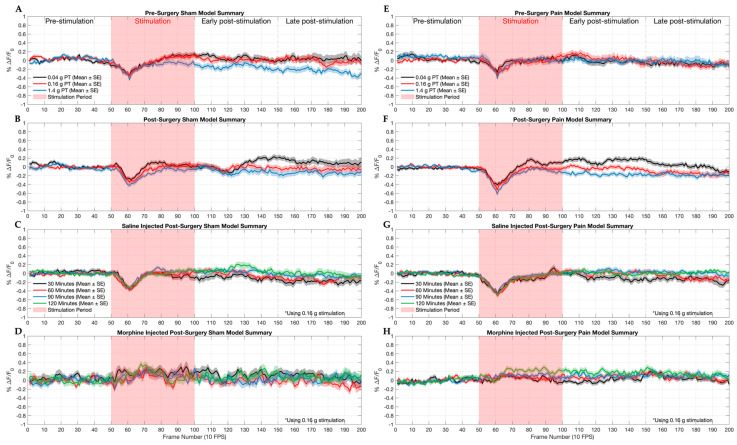
An averaged visualization summary of the percentage change in fluorescence (ΔF/F_0_) in the VTA over time before, during, and after mechanically induced nociceptive stimulation responses resulting from multiple sham-operated mice (n = 4) and partial nerve ligation (PNL) mice (n = 4), with eight animals in total. Each line is an average of 60 stimulations (mean ± SE). (**A**) Decreased VTA activity proportional to the stimulation intensity was observed, with the 1.4 g force stimulation showing the weakest rebound excitation. (**B**) The pain threshold experiment was repeated two weeks after the surgery. A similar response was elicited for each force. The depression in VTA activity is consistent with the pre-surgery pain threshold experiment, and the 1.4 g force response is consistently below baseline. (**C**) A subcutaneous saline injection was administered one day after the post-surgery pain threshold experiment, and the 0.16 g force filament was applied repeatedly. A consistent response was elicited. (**D**) A subcutaneous morphine injection (3.0 mg/kg) was administered one day later. Variability in response is reduced, and the depression is considerably weaker. (**E**) A proportional change in fluorescence respective to the stimulation intensity elicited a clear drop in VTA activity, with the 1.4 g force inducing the most remarkable depression. (**F**) A clear distinction between each stimulation force was observed, with the 1.4 g force stimulation creating sustained difficulty in returning to baseline. (**G**) Upon saline administration, exaggerated depression similar to the 0.16 g force of previous pain threshold experiments was observed due to chronic pain. (**H**) The signature response was nearly eliminated, along with much less variability in response compared to the sham-operated counterpart.

To determine the efficacy of our approach to mapping and monitoring VTA response to nociceptive stimulation within the mesocorticolimbic system, we recorded the VTA’s reaction using fluorescence imaging captured by our µ-CMOS imaging platform in the brains of adult male mice in freely moving conditions. We can elucidate an average response, highlighting the relationship between VTA activity and nociception by interpreting the fluorescence activity associated with acute mechanical stimulations, with individual trace animal data for both cohorts located in the Supplementary Materials, Figures S1–S4. Figure 2A–D depicts the trace response from the sham model mice. Figure 2A shows an apparent response to stimuli, and the fluorescence recovery time indicates the respective stimulus strength, with the 1.4 g force average stimulation making it challenging to return to baseline. Figure 2B provides similar results despite occurring after the sham nerve ligation surgery, showing a successful control across different animals. When applying the 0.16 g force stimulation in Figure 2C, a consistent stimulation response is observed despite the time period following saline administration. The administration of morphine in Figure 2D elicits a stimulation response that is greatly reduced, but the variance in the change in fluorescence increases. Ultimately, the therapeutic effect of morphine can be visualized in real time, summarized across different sham-operated animals.

To accurately investigate our platform’s efficacy and better understand the relationship between chronic pain and acute nociception, we introduced a PNL model cohort, shown in Figure 2E–H, that underwent an identical series of experiments and protocols as its sham-operated counterparts. Note that all the PNL model mice displayed the expected sensitivity patterns consistent with PNL-induced mechanical allodynia. Figure 2E shows a relatively uniform response when experiencing varying intensities of stimulation force. Figure 2F highlights a more distinct separation in the reaction between each stimulation force, with the 0.04 g stimulation force having the greatest rebound excitation response and 1.4 g force stimulation taking the longest to return to baseline with a minor rebound excitation response. A near-uniform response was recorded across all the time points following saline administration, as shown in Figure 2G. Finally, Figure 2H shows a robust reduction in pain response due to the antinociceptive effect of the opioid. Across different animals, we see a similar reaction under antinociceptive conditions, with decreased depression in the activity within the VTA.

**Figure 3:**
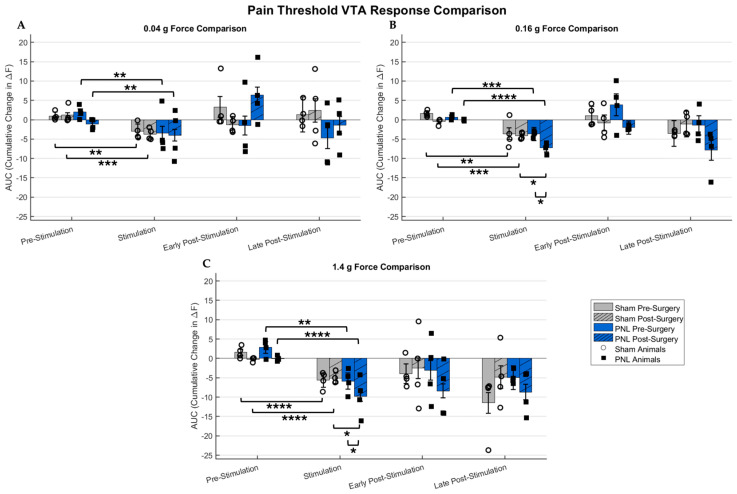
An averaged statistical representation of the area under the curve (AUC) comparing the response of sham-operated and partial nerve ligated (PNL) mice models following exposure to varying stimulation intensities during the pain threshold experiments. Individual points are representative of 15 stimulations averaged per animal, with a total of 60 stimulations for sham (n = 4) mice and 60 stimulations for PNL (n = 4) mice. (**A**) A comparison of the pre- and post-surgery average response for the 0.04 g force stimulation is shown. A statistically significant difference in VTA activity was observed when comparing before and during stimulation prior to and after the respective cohort surgery. (**B**) The 0.16 g force stimulation elicited a more exaggerated depression in the VTA, and an interexperimental statistically significant difference was observed for PNL model mice. Statistical significance was also noted between healthy and diseased animals during stimulation. (**C**) The 1.4 g force realized a much starker response, showing the animals experienced a more intense reaction, and similar statistical significance was noted. Each column represents the mean ± SD of 60 stimuli from four animals. Statistical significance was evaluated using either the appropriate paired t-test or the Wilcoxon Signed-Rank Test following the determination of normality using the Shapiro–Wilk Test. **** denotes *p* < 0.0001, ±SD. *** denotes *p* < 0.001, ±SD. ** denotes *p* < 0.01, ±SD. * denotes *p* < 0.05, ±SD.

**Figure 4:**
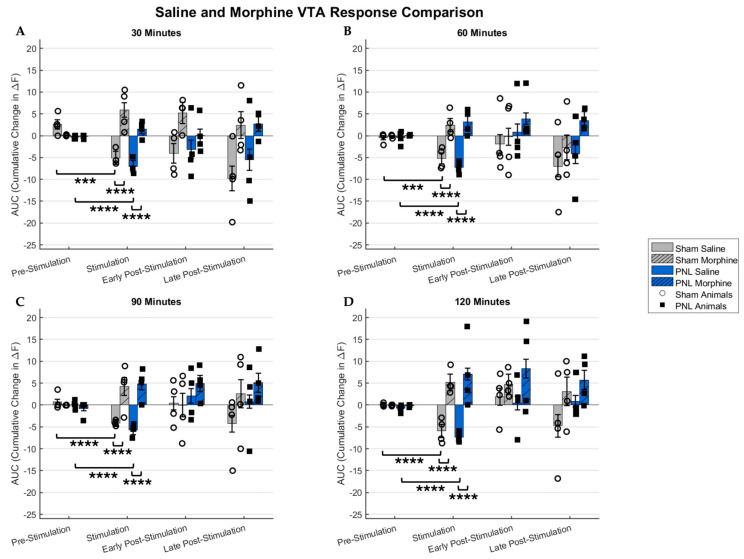
An averaged statistical representation of the area under the curve (AUC) comparing the response of sham-operated and partial nerve ligated (PNL) mice models following exposure to subcutaneous injections of saline and morphine, divided into 30 min blocks. Individual points are representative of 15 stimulations averaged per animal, with a total of 60 stimulations for sham (n = 4) mice and 60 stimulations for PNL (n = 4) mice. (**A**) The 30 min following injection of both saline and morphine, comparing the pre- and post-surgery average responses, is depicted. A statistically significant difference in VTA activity was observed when comparing before and during stimulation. A significant difference in pain response during stimulation after morphine administration was observed. No statistical significance was found between pre-stimulation and stimulation during the morphine experiment (**B**)**.** The average comparative response under the influence of saline and morphine after 60 min. (**C**) The 90 min time point following saline and morphine administration. (**D**) After 120 min, the effects of morphine are still visible, demonstrating a similar response to previous time points. Each column represents the mean ± SD of 60 stimuli from four animals. Statistical significance was evaluated using either the appropriate paired t-test or the Wilcoxon Signed-Rank Test following the determination of normality using the Shapiro–Wilk Test. **** denotes *p* < 0.0001, ±SD. *** denotes *p* < 0.001, ±SD.

To better understand and quantify the severity of the response during the previously mentioned experiments, each stimulation was divided into the following sections: pre-stimulation, stimulation, early post-stimulation, and late post-stimulation. Within each subsection, the respective response correlates to a portion of the stimulation, with a stimulation response evaluation timing being 20 s at 10 frames per second (FPS). In the pre-stimulation subsection, i.e., for the first five seconds, the mice are ideally in a resting state with little to no movement. The mice have not been stimulated, so we expect a stable fluorescence response in the VTA. After five seconds, the mice are mechanically stimulated, with contact being made to the hind paw for five seconds. The fluorescence activity may respond to this stimulation. We then have two post-stimulation subsections lasting five seconds each. These subsections allow for further interpretation of the response as the system attempts to reestablish homeostasis. By calculating the area under the curve every five seconds, comprising 50 frames, and averaging the results, we can statistically analyze the VTA’s response to mechanical nociception under normal and morphine-induced analgesic conditions from each stimulus (60 stimulations averaged from four sham model mice and 60 stimulations from four PNL model mice), as shown in Figure 3 and Figure 4. Further nuances relating to mechanical response can be elicited using these respective subsections.

Figure 3 presents a statistical comparison, using the described method above, to highlight the difference in response between sham model and PNL model mice when exposed to varying forces of mechanical stimulation. It is hypothesized that the pain threshold stimulation response for the sham-operated mice should remain consistent before and after the surgery, as opposed to a marked difference before and after PNL surgery in the pain model cohort, and Figure 3 confirms this hypothesis. Figure 3A highlights the statistically significant depression before and during stimulation with the 0.04 g force von Frey filament before and after the surgery for each respective cohort. For both groups, no statistical significance was identified when comparing the stimulation subsection before and after the surgery, and a statistically significant difference was noted when comparing before and during the stimulation. Figure 3B–C follows a similar trend, with depression increasing as the stimulation force increases, and statistical significance was noted upon stimulation when comparing experimental cohorts. It can be derived that VTA depression and the correlating pain response are exacerbated in PNL model mice. The 0.16 g force in Figure 3B produces a stark response for mice under the influence of chronic pain, as noted by a depression in VTA fluorescence activity, which is significantly stronger following the partial nerve ligation surgery. The most intense stimulation force, as seen in Figure 3C, shows a consistent response. Additionally, there is significant depression in VTA activity when comparing the fluorescence response before and during stimulation before and after surgery for both cohorts. Therefore, the sham surgery was successfully conducted on all the sham-operated animals, as with the nerve ligation procedure for the PNL model mice.

Statistical analysis of therapeutic experiments was also conducted, comparing and highlighting the analgesic effect of morphine on VTA activity in healthy and diseased mice. Figure 4 represents a statistically quantified view of the VTA’s response to pain stimuli, comparing subcutaneous injections of saline and morphine across varying time points. As with the pain threshold response, these averaged stimulations are divided into stimulation subsections. Figure 4A shows that, on average, there is a statistically significant reduction in fluorescence in the VTA during stimulation 30 min after saline injection for the sham and PNL cohorts. We see volatility in response following the morphine injection, but crucially, the signature suppression of VTA activity is not noted. Additionally, there is a statistically significant difference during the stimulation periods following subcutaneous saline and morphine administration. A more consistent response was observed from the PNL model mice. These trends remain consistent across all time points, as shown in Figure 4B–D. No statistical significance was noted when comparing the pre-stimulation and stimulation subsections across all time points under analgesic conditions.

### 2.2. Confirmation of GCaMP6s Virus Expression and CMOS Imaging Device Position

**Figure 5:**
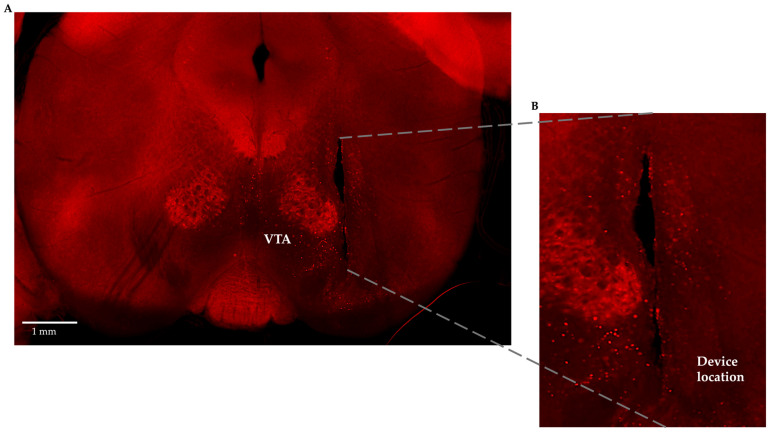
Analysis of the brain was performed following experimentation to verify the location of the CMOS device implantation and the success of the GCaMP6s injection and expression. (**A**) Fluorescence imaging of a representative mouse coronal brain slice following experimentation using an implanted CMOS imaging device. The VTA and device implantation locations are noted. (**B**) A close-up of the implantation area shows the correct location and positive fluorescence. The red signal indicates the nuclear expression of mCherry.

Following the final experiment, the animals underwent perfusion fixation to certify that the results were derived from the positive fluorescence of the VTA due to sufficient GCaMP6s expression. During the terminal procedure, 1 × phosphate-buffered saline (PBS) was irrigated throughout the cardiovascular system to remove excess blood and fluids [18]. A 4% paraformaldehyde solution was used to fix the tissues, preserving the implantation location and preparing the sample for vibratome slicing. Fluorescence microscopy was utilized to identify positive VTA fluorescence and proper implantation location, as observed in Figure 5. Upon analysis, the implantation location suggests successful visualization of the VTA, as the scar left by the device is located at an appropriate depth in the dorsoventral direction with the sensor facing medially. Additionally, positive fluorescence identification in the area implies that the fluorescence obtained by the CMOS imaging platform directly reflects the VTA’s fluorescence activity. The µ-LED of the CMOS imaging device was positioned correctly to provide excitation wavelengths necessary to visualize the VTA’s response to nociceptive stimulation.

## 3. Discussion

### 3.1. CMOS Imaging and Alternative Methods

The deep brain is notoriously difficult to image, especially in smaller animals such as mice with chronic pain conditions, and various methods have been deployed to overcome this challenge [19]. Still, the ability to observe the deep brain during pain processing in real time with freely moving conditions while providing consistent mechanical nociceptive stimulation adds another layer of complexity that must be addressed. To circumvent these limitations, the µ-CMOS imaging platform was selected for its ability to capture fluorescence activity across a defined vertical plane during acute nociceptive stimulation while maintaining minimal invasiveness and compatibility with freely moving conditions. Our µ-CMOS imaging platform is a lightweight, flexible, implantable sensor capable of real-time monitoring of deep brain activity with minimal damage upon implantation due to its thin and malleable design [17]. Due to the adaptable nature of the CMOS device, freely moving conditions are easily implemented and leave the animal nearly uninhibited in terms of movement, which is not practical with traditional fluorescence microscopy and is critical in pain research [20,21]. Unlike fiber photometry, CMOS imaging enables precise spatial mapping of DA release rather than measuring bulk fluorescence intensity alone [22]. Notably, the CMOS imaging device provides a unique perspective regarding deep brain activity by facilitating a vertical viewing area designed to cover the upright span of entire brain regions upwards of 1 mm, unlike that of the gradient refractive index (GRIN) lens [23,24]. This platform offers comprehensive visualization of deep brain fluorescence with impressive spatial and temporal resolution that is not obtainable by electrophysiology and microdialysis, respectively, as seen when investigating freely moving rodent pain models [12,25,26].

### 3.2. Imaging Acute Nociception in a Chronic Pain Model with Therapeutic Treatment

Before investigating morphine’s analgesic interaction with the brain reward circuits, it is crucial to establish the basic response pattern of the VTA when exposed to both chronic and acute pain. To do so, we devised a novel system that adheres to freely moving experimentation, allowing for consistent and accurate hind paw mechanical sensory stimulation via von Frey filaments. The pain threshold assessment is conducted before and two weeks after the respective surgery, evaluating the fluorescence response in terms of the percentage change in fluorescence intensity over time (∆*F*/*F*_0_) across various forces of acute nociception, which is expected to result in a decrease in fluorescence. The stimulations shown are compiled averages over many trials, and the test stimulation forces correlate to the acceptable range used on mice during von Frey hind paw stimulation for testing the nociceptive threshold, with the lightest intensity being 0.04 g force, the medium intensity being 0.16 g force, and the strongest being 1.4 g force [27]. Following the depression in activity, we then hypothesize to observe a rebound excitation event, inversely proportional to the intensity of the stimulation; the more potent the intensity, the weaker the fluorescence rebound excitation response.

The sham-operated and PNL model mice show relatively steady and consistent fluorescence in the fifty frames or five seconds before stimulation occurs across all intensities during the pain threshold experiments pre- and post-surgery. This assists in validating that the sudden drop in fluorescence occurs due to the stimulation experienced upon contact with the von Frey filament. During the stimulation phase, depression in the VTA occurs very rapidly due to efficient and well-structured pain pathways assisted by the fast-acting properties of GABA and other neurotransmitters, followed by a rebound excitation response [28]. For the sham-operated pre- and post-surgery and PNL models pre-surgery, we see a similar stimulation response in all intensities, with more similarity between the 0.04 g and 0.16 g stimulation forces. The 0.04 g force does not reliably induce depression in the VTA in a naive animal and is interpreted as a low-threshold response that does not consistently elicit potential nociceptive responses. Notably, stimulation with 0.04 g force occasionally results in a more prominent rebound excitation in the early and late post-stimulation phases. Lack of sustained depression may be indicative of the brevity of nociceptive inhibition due to it being the lightest of forces. The 0.16 g force appears to produce intermediate suppression and is exacerbated by the presence of mechanical allodynia. The 1.4 g force stimulation is expected to evoke a pain response from naive and neuropathic pain model mice consistently, and this is reflected as this force elicits the most profound depression in ∆*F* and often leads to a weaker rebound excitation for an extended period.

Post-surgery results for the PNL models show a stark separation in response proportional to the intensity of the force. The lightest stimulation force exhibited the most intense rebound excitation and sustained elevation. This is due to the enhanced pain threshold induced by partial sciatic nerve ligation, causing hypersensitivity to sensory stimuli. The animals are more perceptive to this stimulation force, but the evoked nociceptive response is weak and is thought not to incur pain consistently. This dramatically reduces the brain’s natural suppressive response, producing a more intense rebound excitation. The 0.16 g stimulation force falls directly between the lightest and heaviest filaments, further validating that the CMOS imaging platform can observe a distinctive nociceptive response. Finally, the 1.4 g force stimulation elicits a severe depression in VTA activity, heavily reduced excitation rebound, and sustained depression in VTA activity. These findings suggest that this sustained depression occurs due to the stark inhibitory response evoked by such a strong stimulus, exacerbated by allodynia—the lasting pain caused by the stimulation, allowing for a more extended suppressive state. Therefore, the exacerbated VTA response due to sensory stimuli following neuropathic pain surgery potentially indicates that the depression in VTA activity is a response to acute nociception.

Statistical significance was found before and after stimulation within the sham-operated and PNL model mice, positively correlating mechanical stimulation to the respective change in fluorescence due to the area under the curve analysis. The 0.04 g force invoked the most variable shift in response. Across all intensities, interexperimental significance was not noted during the evaluation of the sham-operated responses, signifying successful sham surgery conditions and lending validity to the exacerbated results shown from post-PNL surgery stimulation. Within the PNL model pain threshold experiments, the difference in depression in VTA activity following surgery was significantly more substantial. Based on our findings, it is suggested that the partial nerve ligation surgery effectively induced chronic pain and symptomatically introduced mechanical allodynia. Additionally, we see a consistently increased negative area under the curve during the stimulation as the stimulation force increases.

Signature changes in fluorescence suggest an aversive neuronal response to acute nociceptive stimulation varying in intensity. Therefore, it is crucial to investigate the CMOS imaging platform’s ability to visualize morphine’s antinociceptive properties. As expected, the response following saline injection for the sham models resembled very closely the 0.16 g force response in the pain threshold experiments throughout all time points. A similar trend was followed for the PNL models, but with a greater depression in fluorescence in the VTA. Upon subcutaneous administration of morphine, a marked difference in VTA response was observed, including a stark reduction in depression in the VTA upon stimulation for the sham-operated mice and a near elimination of response for the PNL model mice. It can be inferred that the response seen due to stimulation is nociceptive in nature, based on the attenuated response following morphine administration. However, the sham-operated mice exhibited more variation in response to stimulation than the PNL mice. Subjects experiencing chronic pain presented a very consistent response. This may be attributed to the effects of chronic pain over time on the mesocorticolimbic system and its ability to alter its function fundamentally [29]. Chronic pain is known to naturally place the dopaminergic neurons in a state of consistent attenuation, even when uninhibited, leaving the system’s potential range for variation severely limited, slowing down the entire neural circuit. Despite this, our results indicate a reversal of VTA activity suppression due to morphine’s µ-opioid-binding biochemical mechanism, allowing for a therapeutically induced analgesic state.

We observed strong statistical significance across all the groups when comparing before and during stimulation within the saline injection experiment, providing results similar to the pain threshold protocol. The antinociceptive effect of morphine is also present when statistically observing the change in fluorescence across all groups. For the PNL model mice, the statistical significance is much greater than that of the sham-operated groups, which aligns with the expected result due to the presence of allodynia. Although these findings provide strong evidence of nociceptive modulation of the VTA due to mechanical acute stimulation, fluorescence imaging alone may not fully capture the behavioral and subjective scope of chronic neuropathic pain and the effects of antinociceptive therapeutics. Complementary behavioral assays may prove beneficial, and their incorporation is a focus for future studies. Additionally, the method for calculating the change in fluorescence over time, as detailed in the results and methods sections, specifically filters the potentially confounding basal excitatory effects of morphine. Therefore, future studies incorporating baseline tracking for pharmacological dissection may provide additional context, as demonstrated in previous studies [15]. While the feasibility of imaging morphine-induced modulation of the VTA is established in this study, we plan to design future studies incorporating opioid antagonist controls such as naloxone or naltrexone to validate that the observed effects are mediated via opioid receptor pathways [30]. Finally, given the technical complexities of our µ-CMOS imaging platform, longevity of experiments, and surgical procedures, the number of animals per cohort was limited. While consistent and statistically significant effects were observed, these preliminary conclusions would further benefit from the use of a larger sample size to confirm and generalize our findings.

## 4. Materials and Methods

### 4.1. Ethics Statement and Animal Care

All the experimental protocols and surgical procedures were approved by the Animal Care Committee of the Graduate School of Pharmaceutical Sciences of Teikyo University and conducted with respect to the guidelines of the National Institutes of Health and the Japanese Pharmacological Society (approval number: 23-030). This study included male C57BL/6 mice (6 weeks old; SLC Japan, Shizuoka, Japan), with the results deriving from eight animals (four sham and four PNL models). All the mice were separated into individual cages and resided in a room maintained at 23 ± 2 °C with an alternating 12 h light–dark cycle and had food and water ad libitum.

### 4.2. Experimental Design

**Figure 6:**
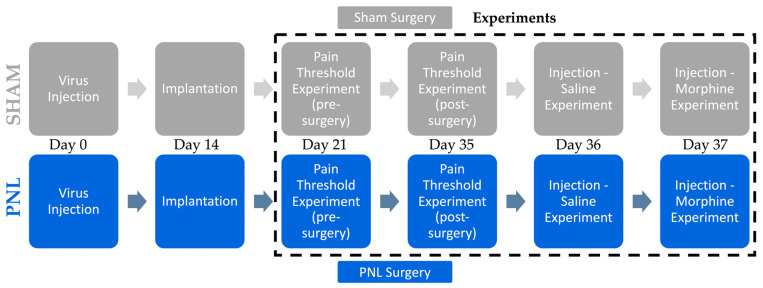
Surgical and experimental timeline for both sham-operated and partial sciatic nerve ligation (PNL) animal groups. A strict timeline was enforced to ensure identical experimental conditions. Virus injection occurred first, followed by a two-week waiting period. The implantation of the CMOS imaging device took place with one week of healing post-surgery. The pre-surgery pain threshold experiment was performed to establish a baseline pain response via fluorescence activity of the VTA. Immediately following the experiment’s conclusion, the respective sham or PNL surgery was performed. After a two-week waiting period, the pain threshold experiment was repeated with identical conditions to determine if the VTA response differed. With a single day in between, the saline injection and morphine injection experiments were conducted to evaluate the therapeutic properties of morphine.

The experimental design was created to allow identical conditions for all the animals, as shown in Figure 6. The animals underwent adeno-associated virus (AAV) microinjection and were allowed two weeks for virus expression. The CMOS imaging device was then implanted into the VTA area, and the subjects were allowed one week for recovery. Next, the animals underwent the initial mechanical nociceptive threshold measurement while recording the fluorescence activity of the VTA. During this experiment, the animal was stimulated repeatedly, with at least 30 s between each stimulation. Immediately following the initial pain threshold experiment, the respective sham or PNL surgery was performed. The animal was allowed to develop neuropathic pain for two weeks, and an identical mechanical nociceptive threshold measurement was conducted. The following day, the animal was administered a subcutaneous injection of saline or morphine and stimulated repeatedly using the 0.16 g force filament, sectioned into 30 min segments. An identical injection protocol using morphine, with a dosage of 3.0 mg/kg, was performed on the next and final day. Each experiment was recorded using the newest CIS-OS analysis hardware and software, elaborated on in our previous study [15].

### 4.3. Imaging Device and Fabrication

**Figure 7:**
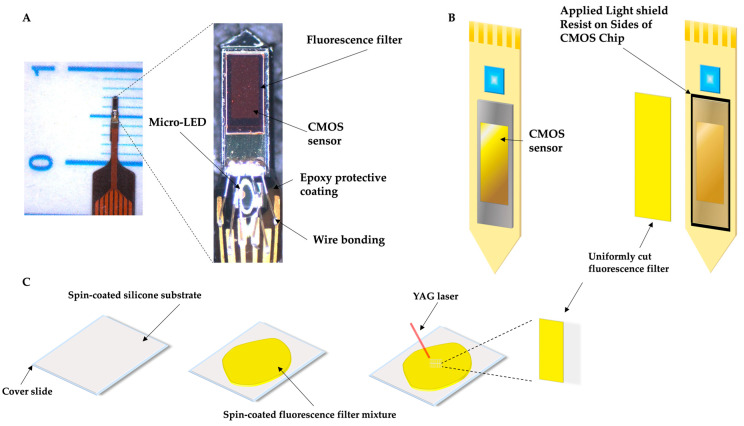
The µ-CMOS imaging fabrication process, adapted from our recent paper [15]. (**A**) On the left, the custom-designed CMOS imaging device is shown, featuring a CMOS chip measuring 450 µm × 1275 µm and an imaging area of 300 µm × 675 µm. (**B**) The µ-LED and CMOS chip first adheres to the flexible substrate, and the hand-fabricated filter is thermo-vacuumed to the imaging surface. We apply a black light shield resist along the sides of the CMOS imaging device to interrupt excess light and an epoxy coating for hardware protection. (**C**) Spin-coating is used on a glass slide to create a smooth silicone substrate layer for the fluorescence filter, and the fluorescence filter mixture is spin-coated on the new foundation and baked. To produce identical, uniformly cut fluorescence filters, a YAG laser is implemented.

The implantable µ-CMOS imaging device, shown in Figure 7, was designed and fabricated at the Nara Institute of Science and Technology (NAIST) for rodent brain imaging. The device was created using a needle-shaped design and flexible printed circuit (FPC) substrate (Taiyo Technolex Co., Ltd., Wakayama, Japan) to enable access to the deep brain while minimizing extraneous damage to neuronal tissue. The device utilizes the µ-CMOS imaging sensor (TSMC, Hsinchu, Taiwan) and the µ-LED (ES-VEBCM12A, Epistar Corporation, Hsinchu, Taiwan), which has a length of 280 µm by 305 µm and emits a wavelength of 460 nm, at the head of the device to maximize imaging depth, allowing the excitation of previously injected GCaMP6s AAV. The CMOS imaging area is arranged rectangularly to allow the complete viewing of the neuronal structure. It consists of a 40 × 90 array of pixels, each pixel being 7.5 µm by 7.5 µm. This layout assists in visualizing the entirety of the VTA and provides a unique vertical imaging area, notoriously challenging to achieve in deep-brain fluorescence imaging. A unique process of filter fabrication, mounting of the µ-LED and µ-CMOS sensor, and Parylene-C coating for biocompatibility is required for successful experimental implementation and is detailed further in previous publications [14,15].

### 4.4. Stereotaxis Surgery

Each mouse underwent three surgeries: (i) GCaMP6s AAV injection, (ii) CMOS imaging device, and (iii) either sham or partial ligation of the sciatic nerve surgery. The general surgical protocols for AAV injection and CMOS imaging implantation are discussed at length in our previous publications [13,14,15,31].

### 4.5. GCaMP6s AAV Injection

The AAV9-hSyn-GCaMP6s-nls-mCherry AAV was injected into the VTA of adult mice to achieve virus-mediated expression of GCaMP6s. This genetically engineered calcium indicator can assist in accurately visualizing the dynamics of the VTA when sufficiently stimulated using the appropriate excitation wavelength. The virus was injected into the VTA at 0.15 µL/min for 5 min, requiring a minimum of 14 days to develop full expression.

Each animal was anesthetized using 1.5–2.0% vaporized isoflurane and fixed stereotaxically (Narishige, Tokyo, Japan) with appropriate head mounting fixtures. The animal was evaluated to ensure an unconscious state. An incision was made along the sagittal suture, exposing the skull. The surface of the skull was cleaned, and the height of the bregma and lambda was adjusted to be even. Starting with bregma, the location of the burr hole was measured and marked above the VTA. The burr hole was created, and the dura was broken. The location of the injection was as follows (calculated from the literature): anteroposterior (AP): −3.2 mm, mediolateral (ML): −0.6 mm, and dorsoventral (DV): −4.55 mm. The AAV solution was administered using a glass capillary needle loaded with the GCaMP6s AAV, which was slowly inserted into the VTA of the mouse. Following the successful release of the virus, the needle remained within the brain for an additional 5 min to ensure complete expulsion of the virus into the surrounding tissue. The needle was then carefully removed and discarded as appropriate. The incision was sutured closed, and the animal was monitored closely until it was awake and moving as expected. The animal was then moved back to the respective home cage and monitored for the following weeks.

### 4.6. CMOS Imaging Device Implantation

After the two weeks required for sufficient viral expression, the CMOS imaging device was implanted in the VTA using the identical coordinates in the injection surgery protocol. The animal was anesthetized with 1.5–2.0% isoflurane, and the head was fixed into the stereotaxic surgery apparatus. A surgical protocol similar to the AAV injection was utilized to locate the VTA. To better suit the shape of the imaging device, an oval-shaped burr hole was created directly above the target location. Stainless steel screws were then strategically mounted around the burr hole to ensure the stability and longevity of the implantation, reducing the risk of detachment. The device was lowered until the bottom of the sensor was even with the brain’s surface to ensure accurate implantation depth. It was then slowly inserted to ensure no bending of the device and to minimize damage to the surrounding tissues. After reaching the appropriate depth, the device was fixed using dental cement incorporated with powdered carbon (Sigma-Aldrich, St. Louis, MO, USA). Due to the CMOS sensor’s sensitivity to erroneous light, the carbon powder assists in providing consistent lighting conditions. After CMOS sensor implantation, the animal was monitored until waking to ensure healthy behavior. The top of the device was protected using parafilm, and the animal was placed back into its respective home cage.

### 4.7. Sham and Partial Ligation of the Sciatic Nerve Surgery

Two experimental groups, sham-operated and partial sciatic nerve ligation models, were created to properly differentiate between chronic pain and healthy VTA response. To perform partial sciatic nerve ligation, the mouse was anesthetized with 1.5–2.0% isoflurane and positioned so that the left hind leg was easily accessible. The skin resting above the femur was shaved, and an incision was created approximately 1.5 cm long on the lateral thigh. The superficial muscle was bluntly dissected using fine forceps to expose the underlying sciatic nerve. Upon visualization and confirmation of the nerve, 8–0 silk suture was inserted through approximately 1/3 to 1/2 of the nerve and ligated. The muscle was then sutured closed, followed by the skin. The animal was monitored until awake and then placed back into the respective home cage. The same protocol was implemented for the sham-operated models, but the sciatic nerve was only exposed and not ligated. Therefore, the sciatic nerve remained completely intact in the sham-operated model.

### 4.8. Imaging and ROI Analysis

We initially began our study by evaluating our platform using sham-operated mice undergoing a series of experiments to determine the VTA’s response to acute nociceptive stimulation in a healthy model and then compared the response to a chronic pain PNL cohort. All the animals underwent a pain threshold measurement before receiving sham or PNL surgery to determine the baseline response of the VTA to varying forces of stimuli, including 0.04 g, 0.16 g, and 1.4 g force von Frey filaments. Immediately following baseline measurements, the respective sham or PNL surgery was performed. After two weeks, the same pain threshold analysis was conducted to assess if any change in response occurred. One day later, an injection experiment was performed where saline was administered subcutaneously, and the subjects were stimulated using the 0.16 g force von Frey filament repeatedly. After one final day, morphine was administered following the same injection experimental protocol. Using different stimulation intensities, antinociceptive alterations, and treatment groups, we can understand how the depression in fluorescence in the VTA, visualized with the AAV-derived GCaMP6s, is related to adverse stimuli.

The CIS-OS platform allows for the analysis of customizable ROIs. Using the CIS-OS analysis software, 10 × 10 pixel regions were selected based on the implantation location and strength of fluorescence. We first applied the percentage change in fluorescence intensity over time:

$$\frac{∆F}{F_{0}}\%=\frac{F-F_{0}}{F_{0}}\times 100$$

where *F* is the fluorescence intensity, and *F*_0_ is the baseline fluorescence. Summarizing trace data comparing the sham-operated animal response to the PNL model response is depicted in Figure 2, showing the ∆*F*/*F*_0_ of the VTA using a hand-selected 10 × 10 pixel region of interest (ROI), correlating to nociceptive stimulation. Each stimulation intensity or time period is an average of stimulations, showing the change in fluorescence intensity over time (15 stimulations averaged from one animal, with four animals in each group, resulting in 60 stimulations in total). Additionally, every 50 frames, around 5 s, is segmented to distinguish how the stimulation influences the response. To ensure the accurate interpretation of the fluorescence changes resulting from mechanical stimulation perturbations, particularly when animals are under the influence of morphine, the fluorescence signal normalization is tailored to match the experimental method. We have employed a widely utilized normalization method for relative fluorescence modulation of stimulation responses [12,13,14,15,16,32]. In this regard, *F*_0_ is updated with respect to each individual stimulation, with the first 50 frames acting as the baseline fluorescence. The following 200 frames, encapsulating *F*, are calculated in reference to the respective pre-stimulation fluorescence. This dynamic baseline minimizes the confounding effects from fluorescence degradation, movement artifacts, and the effects of morphine’s known tonic excitation of VTA dopaminergic neurons, isolating the phasic response to mechanical stimulation [33].

### 4.9. Implantation Site Confirmation

Upon completing the final experiment, each animal underwent a terminal perfusion procedure to clean and fix the brain, preparing for AAV and implantation site confirmation. The animal was sedated, and the thoracic cavity was opened to expose the heart. A blunt needle was inserted into the left ventricle, allowing a flow of PBS, and the aorta was severed. A 4% paraformaldehyde (PFA, Thermo Fisher Scientific, Waltham, MA, USA) solution was perfused throughout the system to fix the brain. After being sufficiently treated, the brain was removed and stored. A VT1200 semiautomatic vibrating blade vibratome (Leica, Deer Park, IL, USA) was used to create 100 µm thick slices of each brain. The slices were placed on slides and analyzed using fluorescence microscopy.

### 4.10. Statistical Analysis

Statistical significance was evaluated using either the appropriate paired *t*-test or the Wilcoxon Signed-Rank Test following the determination of normality using the Shapiro–Wilk Test. A *p*-value of 0.05 was considered statistically significant, and all data are represented as mean ± standard error.

## 5. Conclusions

In this study, we have shown the efficacy of this platform for visualizing the relationship between VTA fluorescence response and acute nociception in the deep brains of freely moving adult mice, shifting the capabilities to a new field of research. By applying varying sensory stimulation forces, we elicited a proportional response in the depression in VTA exacerbated by chronic pain, which is reversed upon morphine administration.

## Data Availability

The data supporting this study’s findings are available from the corresponding author upon request.

## Supplementary Materials

The following supporting information can be downloaded at: https://www.mdpi.com/article/10.3390/ijms26136526/s1.

## Abbreviations

The following abbreviations are used in this manuscript:

DA	Dopamine
VTA	Ventral tegmental area
NAc	Nucleus accumbens
GABA	γ-aminobutyric acid
PNL	Partial nerve ligation
PFC	Prefrontal cortex
CMOS	Complementary metal–oxide–semiconductor
CRF	Corticotropin-releasing factor
AAV	Adeno-associated virus
ROI	Region of interest
CNS	Central nervous system
