# Supplementary material for: Mapping Morphine’s Antinociceptive Impact on the Ventral Tegmental Area During Nociceptive Stimulation: A Novel Microimaging Approach in a Neuropathic Pain Model
Source: Int J Mol Sci. 2025 Jul 7;26(13):6526. doi: 10.3390/ijms26136526 (PMC12250161; doi:10.3390/ijms26136526)
Supplement: Supplementary file 1 [file ijms-26-06526-s001.zip › ijms-3725115-supplementary.pdf]

Figure S1

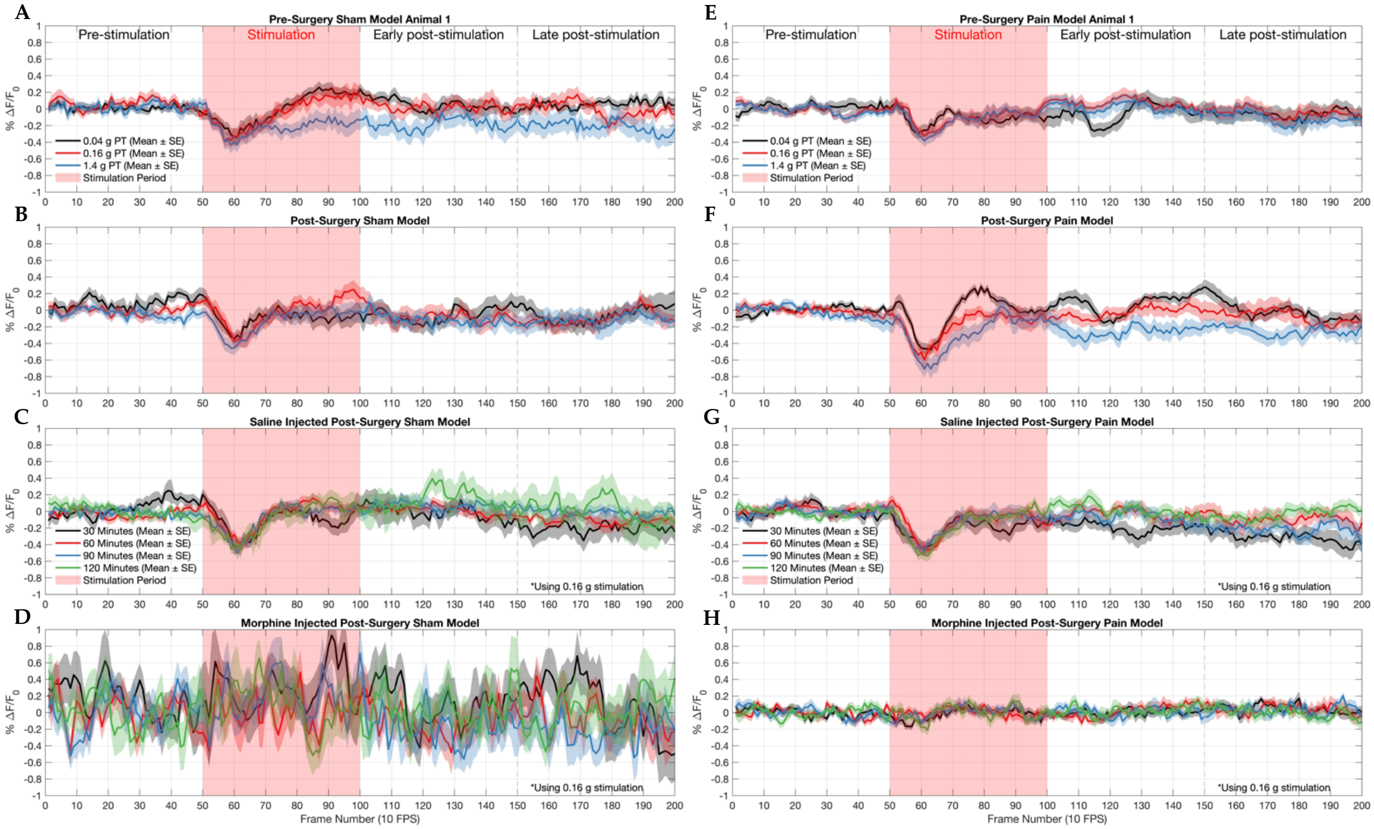

Figure S2: Sham Animal 1 and PNL Animal 1's averaged visualization of the percentage change in fluorescence ($\Delta F/F_0$) in the VTA over time before, during, and after mechanically-induced stimulation responses (n=1 per group). Each line is an average of 15 stimulations. **(A)** Pain threshold (PT) experiment fluorescence response prior to surgery, using three nociceptive stimulations: 0.04, 0.16, and 1.4 g for the sham animal. **(B)** The PT experiment was repeated two weeks after receiving the sham surgery. **(C)** A subcutaneous saline injection was administered one day after the post-surgery PT experiment, and the 0.16 g force filament was applied repeatedly. **D)** A subcutaneous morphine injection was administered one day later. **(E)** Pain threshold (PT) experiment fluorescence response prior to surgery, using three nociceptive stimulations for the PNL animal. **(F)** The PT experiment was repeated two weeks after receiving the PNL surgery. **(G)** A subcutaneous saline injection was administered one day after the post-surgery PT experiment, and the 0.16 g force filament was applied repeatedly. (**H)** A subcutaneous morphine injection was administered one day later.

Figure S2

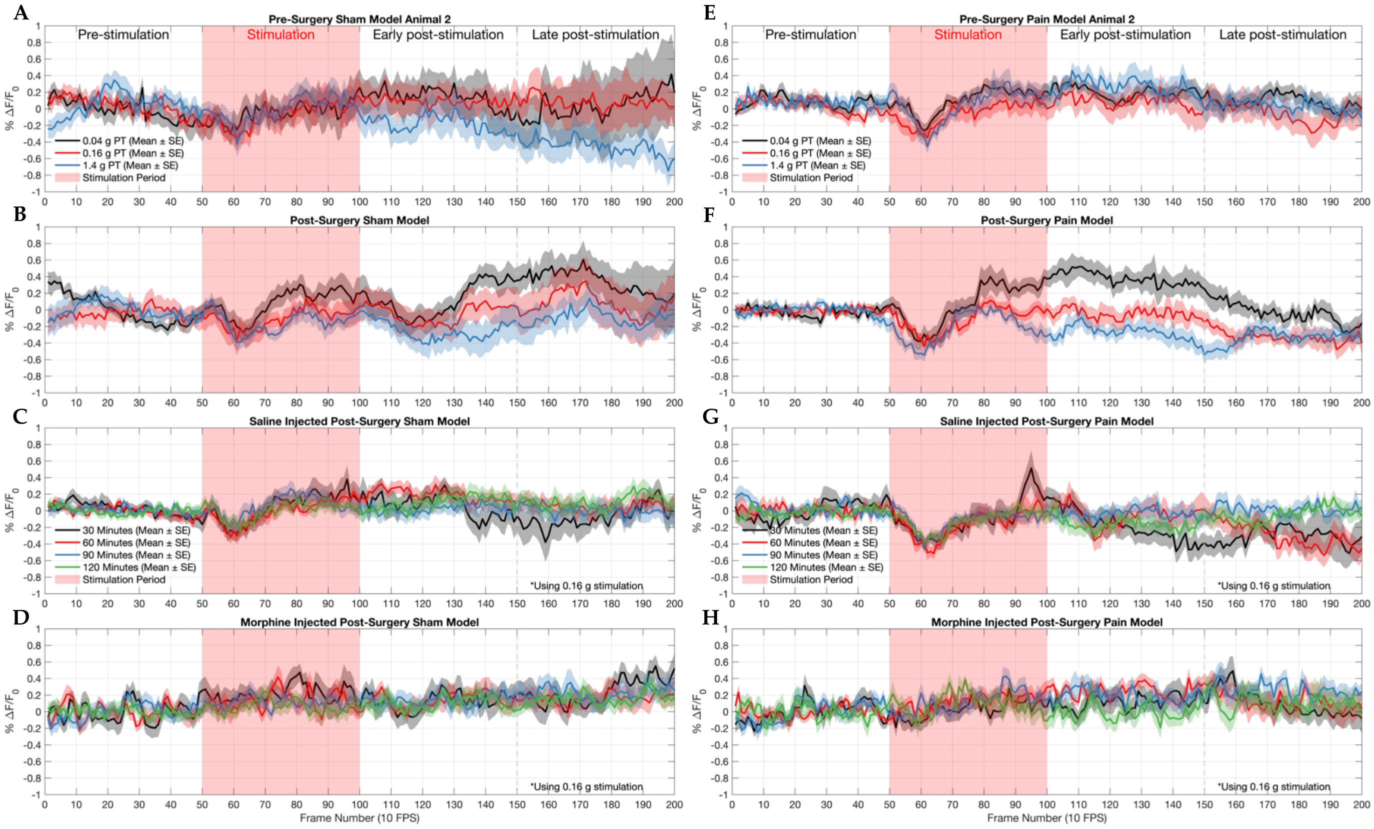

Figure S2: Sham Animal 2 and PNL Animal 2's averaged visualization of the percentage change in fluorescence ($\Delta F/F_0$) in the VTA over time before, during, and after mechanically-induced stimulation responses (n=1 per group). Each line is an average of 15 stimulations. **(A)** Pain threshold (PT) experiment fluorescence response prior to surgery, using three nociceptive stimulations: 0.04, 0.16, and 1.4 g for the sham animal. **(B)** The PT experiment was repeated two weeks after receiving the sham surgery. **(C)** A subcutaneous saline injection was administered one day after the post-surgery PT experiment, and the 0.16 g force filament was applied repeatedly. **D)** A subcutaneous morphine injection was administered one day later. **(E)** Pain threshold (PT) experiment fluorescence response prior to surgery, using three nociceptive stimulations for the PNL animal. **(F)** The PT experiment was repeated two weeks after receiving the PNL surgery. **(G)** A subcutaneous saline injection was administered one day after the post-surgery PT experiment, and the 0.16 g force filament was applied repeatedly. (**H)** A subcutaneous morphine injection was administered one day later.

Figure S3

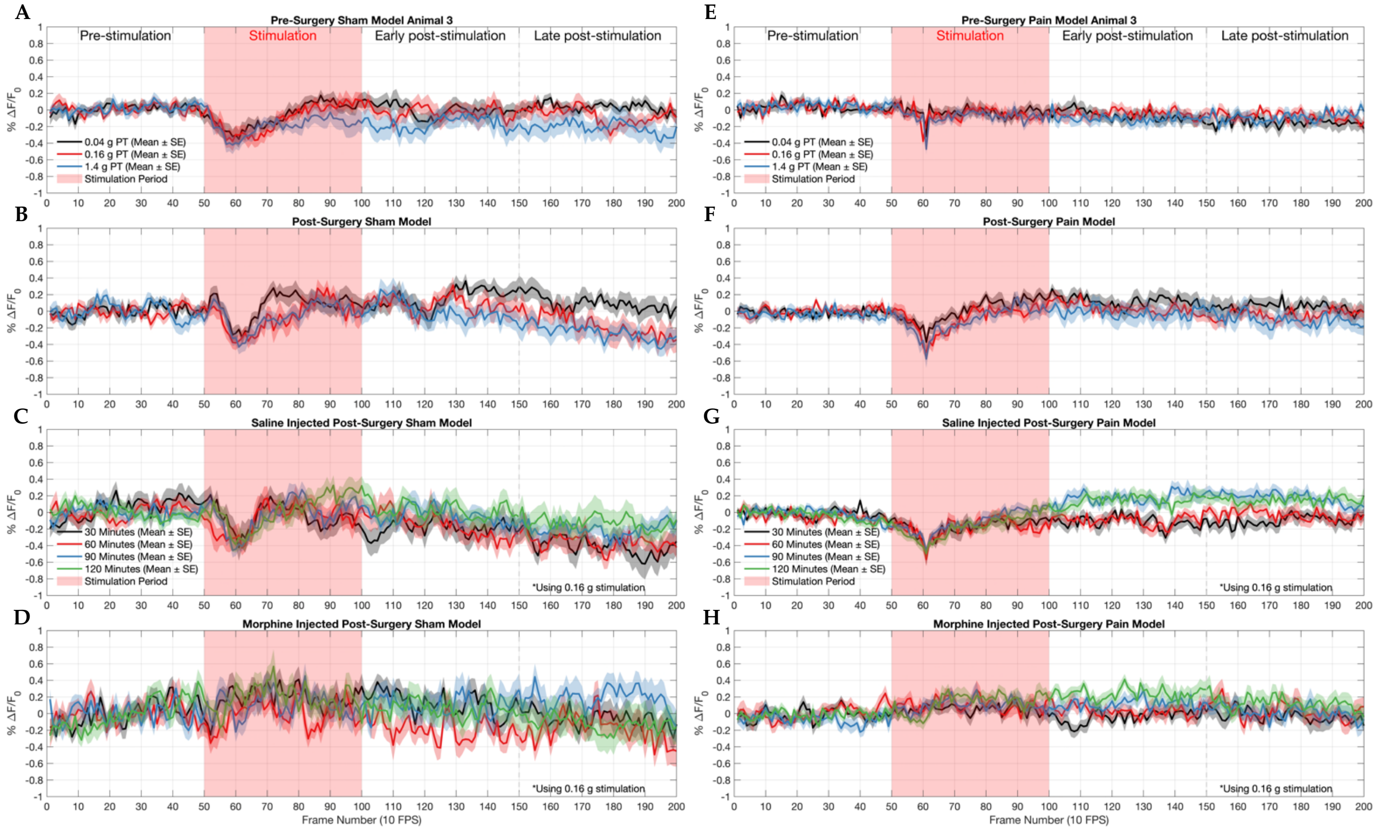

Figure S3: Sham Animal 3 and PNL Animal 3's averaged visualization of the percentage change in fluorescence ($\Delta F/F_0$) in the VTA over time before, during, and after mechanically-induced stimulation responses (n=1 per group). Each line is an average of 15 stimulations. **(A)** Pain threshold (PT) experiment fluorescence response prior to surgery, using three nociceptive stimulations: 0.04, 0.16, and 1.4 g for the sham animal. **(B)** The PT experiment was repeated two weeks after receiving the sham surgery. **(C)** A subcutaneous saline injection was administered one day after the post-surgery PT experiment, and the 0.16 g force filament was applied repeatedly. **D)** A subcutaneous morphine injection was administered one day later. **(E)** Pain threshold (PT) experiment fluorescence response prior to surgery, using three nociceptive stimulations for the PNL animal. **(F)** The PT experiment was repeated two weeks after receiving the PNL surgery. **(G)** A subcutaneous saline injection was administered one day after the post-surgery PT experiment, and the 0.16 g force filament was applied repeatedly. (**H)** A subcutaneous morphine injection was administered one day later.

Figure S4

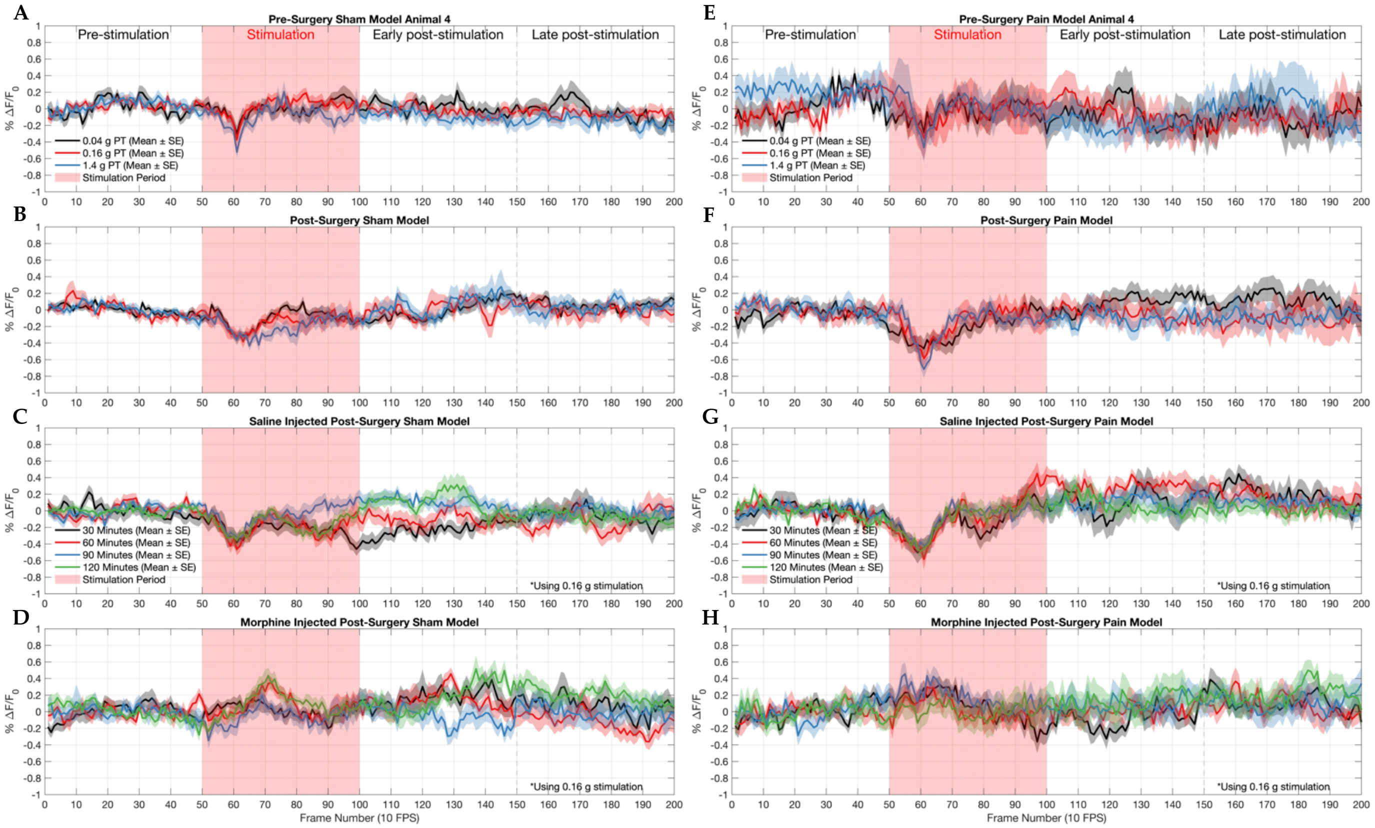

Figure S4: Sham Animal 4 and PNL Animal 4's averaged visualization of the percentage change in fluorescence ($\Delta F/F_0$) in the VTA over time before, during, and after mechanically-induced stimulation responses (n=1 per group). Each line is an average of 15 stimulations. **(A)** Pain threshold (PT) experiment fluorescence response prior to surgery, using three nociceptive stimulations: 0.04, 0.16, and 1.4 g for the sham animal. **(B)** The PT experiment was repeated two weeks after receiving the sham surgery. **(C)** A subcutaneous saline injection was administered one day after the post-surgery PT experiment, and the 0.16 g force filament was applied repeatedly. **D)** A subcutaneous morphine injection was administered one day later. **(E)** Pain threshold (PT) experiment fluorescence response prior to surgery, using three nociceptive stimulations for the PNL animal. **(F)** The PT experiment was repeated two weeks after receiving the PNL surgery. **(G)** A subcutaneous saline injection was administered one day after the post-surgery PT experiment, and the 0.16 g force filament was applied repeatedly. **(H)** A subcutaneous morphine injection was administered one day later.
